# Retrospective cell lineage reconstruction in humans by using short tandem repeats

**DOI:** 10.1016/j.crmeth.2021.100054

**Published:** 2021-07-26

**Authors:** Liming Tao, Ofir Raz, Zipora Marx, Manjusha S. Ghosh, Sandra Huber, Julia Greindl-Junghans, Tamir Biezuner, Shiran Amir, Lilach Milo, Rivka Adar, Ron Levy, Amos Onn, Noa Chapal-Ilani, Veronika Berman, Asaf Ben Arie, Guy Rom, Barak Oron, Ruth Halaban, Zbigniew T. Czyz, Melanie Werner-Klein, Christoph A. Klein, Ehud Shapiro

**Affiliations:** 1 Department of Computer Science and Applied Mathematics, Weizmann Institute of Science, Rehovot 761001, Israel; 2 Department of Dermatology, Yale University School of Medicine, New Haven, CT 06520-8059, USA; 3 Experimental Medicine and Therapy Research, University of Regensburg, Franz-Josef-Strauß-Allee 11, 93053 Regensburg, Germany; 4 Division of Personalized Tumor Therapy, Fraunhofer Institute for Experimental Medicine and Toxicology Regensburg, Am Biopark 9, 93053 Regensburg, Germany

**Keywords:** single-cell genomics, cell lineage reconstruction, molecular inversion probes, short tandem repeats

## Abstract

Cell lineage analysis aims to uncover the developmental history of an organism back to its cell of origin. Recently, novel *in vivo* methods utilizing genome editing enabled important insights into the cell lineages of animals. In contrast, human cell lineage remains restricted to retrospective approaches, which still lack resolution and cost-efficient solutions. Here, we demonstrate a scalable platform based on short tandem repeats targeted by duplex molecular inversion probes. With this human cell lineage tracing method, we accurately reproduced a known lineage of DU145 cells and reconstructed lineages of healthy and metastatic single cells from a melanoma patient who matched the anatomical reference while adding further refinements. This platform allowed us to faithfully recapitulate lineages of developmental tissue formation in healthy cells. In summary, our lineage discovery platform can profile informative somatic mutations efficiently and provides solid lineage reconstructions even in challenging low-mutation-rate healthy single cells.

## Highlights

• A human cell lineage discovery platform based on somatic STR mutations
• The platform has been validated with a high resolution of *ex vivo* cell lineage tree
• A melanoma lineage tree inferred from STR agrees with sampling location
• Lineage trees of healthy cells support separation of embryonic germ layers

## Motivation

Human cell lineage trees can provide answers to the most profound open questions in human biology and medicine. Obtaining this knowledge is a long journey worth taking. We pioneered the concept, the mathematical foundations, and the implementation of the human cell lineage discovery method utilizing naturally acquired microsatellite mutations by individual cells, to reconstruct trees among human cells. We kept improving its power. From 128 microsatellites per cell to 2,000, we have now reached a panel of over 50,000 loci, barcoded and sequenced cost effectively. Retrieving this unprecedented amount of microsatellite data would enable precise cell lineage tree reconstruction.

## Graphical abstract

**Figure:**
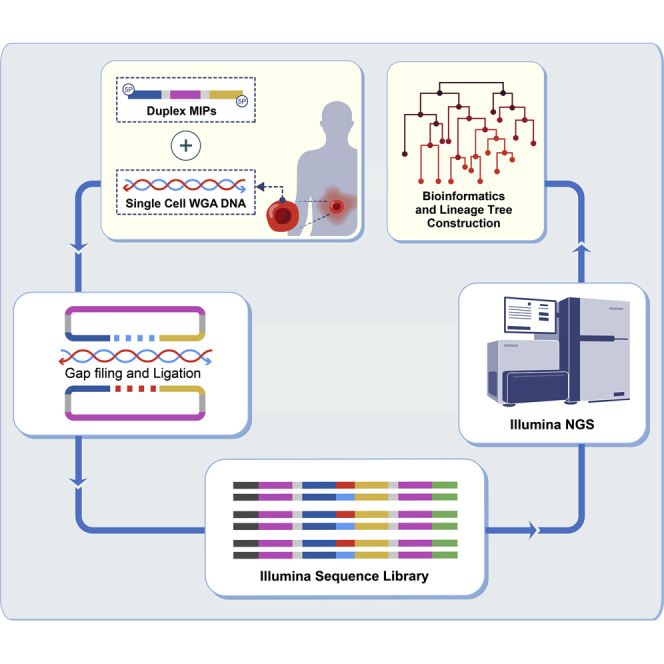

## Teaser

Tao et al. present a biological-computational cell lineage discovery platform that enables efficient acquisition of tens of thousands of short tandem repeat targets in human single cells and tailored cell lineage reconstruction with its integrated bioinformatic pipeline and database management system.

## Introduction

Many fundamental open questions in human biology and medicine can be answered if the structure and dynamics of the human cell lineage tree are uncovered (Chapal-Ilani et al., 2013; Tang, 2020; Wagner and Klein, 2020). Yet *Caenorhabditis elegans* remains the only organism with a known cell lineage tree (Sulston and Horvitz, 1977; Sulston et al., 1983), whereas, more recently, high-throughput molecular-recording cell lineage methods have been applied to model cell lineages in model organisms such as zebrafish and mouse (Frieda et al., 2017; Kalhor et al., 2017; McKenna et al., 2016; Raj et al., 2018; Schmidt et al., 2017; Spanjaard et al., 2018; Wagner and Klein, 2020). All these studies demonstrate how valuable cell lineage information can be when done at scale and with tunable mutation rates. In contrast, the reconstruction of human cell lineages has to rely on somatic mutations occurring naturally during cell divisions, such as L1 retro transposition event, copy number variants (CNVs), single nucleotide variants (SNVs), mitochondrial heteroplasmy (Ludwig et al., 2019) and short tandem repeats (STRs) (Behjati et al., 2014; Evrony et al., 2012, 2015; Frumkin et al., 2005, 2008; Lodato et al., 2015; Mann et al., 2016; Salipante and Horwitz, 2006). Among those, STRs are the largest contributors of *de novo* mutations and are highly abundant (Woodworth et al., 2017). Moreover, STRs' mutation rates are predictable as they correspond to the type of the repeating unit and number of repeats (Willems et al., 2014). Therefore, targeting a selection of STR loci is an effective method for tapping into a rich source of somatic mutations. However, existing methods for accurate genotyping of STRs, such as the AccessArray-based platform (AA pipeline) (Biezuner et al., 2016), remain limited in their throughput or single-cell (SC) compatibility (Table S2 previous protocols). To enable human cell lineage tracing, we strive for (1) improving STR profiling of SCs; (2) validating our lineage tree algorithms by using an experimental setup with known ground truth; (3) comparing generated lineage trees with current state-of-the-art results from SNV profiling; and (4) exploring the power of the approach for healthy SCs from different human cell lineages.

## Results and discussion

**Figure 1:**
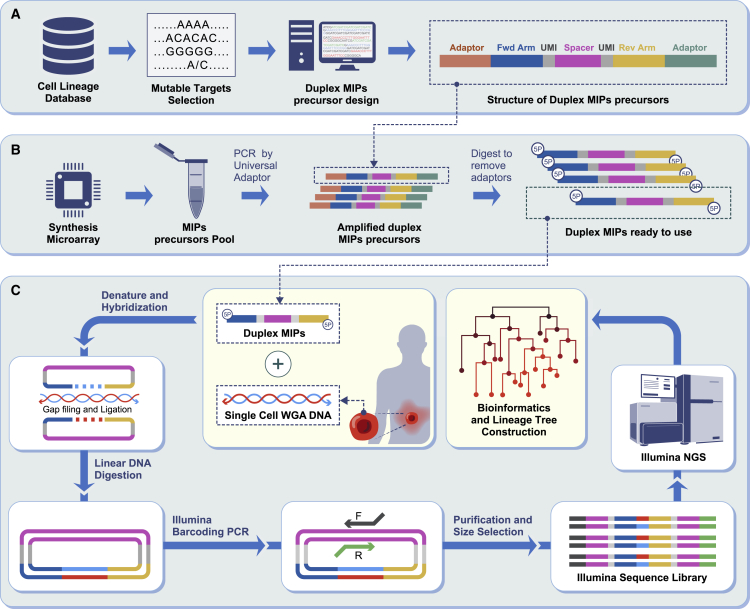
Duplex MIPs-based cell lineage workflow (A) Design of duplex MIPs precursor: desired targets are selected from our cell lineage database and precursors are designed. (B) Duplex MIPs preparation: duplex MIPs precursors are synthesized on a microarray, pooled and amplified by PCR. The product is digested by MlyI to remove the universal adaptors (orange and green), purified and diluted to obtain active duplex MIPs. (C) Duplex MIPs and template DNA are mixed together to allow annealing of the targeting arms (blue and yellow) to the regions flanking the targeted sequence in the template DNA, the products are then circularized by gap filling, facilitated by DNA polymerase and ligase. Linear DNA, including excess MIPs and template DNA, is eliminated by exonucleases digestion. The Illumina sequencing library is generated individually for each sample in a PCR step, facilitating addition of adaptors and barcodes. Libraries are pooled and sequenced by using Illumina next-generation sequencing platform, followed by analysis of the raw reads to detect mutations. The latter are then used to infer the cell lineage tree.

We tested molecular inversion probes (MIPs; also termed padlock probes) for high-throughput and cost-effective cell lineage tracing. MIPs are single-strand DNA molecules composed of two distal targeting arms connected with an internal linker region, which have the potential for much higher multiplexing capability and better specificity compared with multiplex PCR (Nilsson et al., 1994). Several studies have optimized this approach for use with various informative markers (Cancer Genome Atlas Research et al., 2013; Shen et al., 2011) (Kosuri and Church, 2014) (Vos et al., 2015). MIPs have been shown to allow successful capture of tri- and hexa-nucleotide STRs at a low scale, 102 loci per reaction (Carlson et al., 2015). We developed our STRs targeting platform with duplex MIPs produced from precursors synthesized on high-resolution microarray resulting in the capture of ∼12,000 STR targets. Briefly, MIP precursors for selected STR loci (hyper-mutable STRs are discussed later) and optional patient-specific areas of interest were designed, synthesized on a microarray (Figure 1A, duplex MIPs structure is discussed in STAR Methods), and processed to duplex MIPs (Figure 1B, see section “duplex MIPs preparation” in “STAR Methods). Targeted enrichment of each sample is performed in a series of reactions: hybridization between the duplex MIPs and the template DNA, gap filling, ligation, followed by nuclease digestion (Figure 1C, see section “duplex MIPs-based targeted enrichment pipeline” in STAR Methods). Sequencing libraries are prepared by PCR, with primers that contain a unique Illumina barcode combination for each sample, then are pooled and sequenced prior to analysis and cell lineage reconstruction (Figure 1C). Pipeline details are mentioned in STAR Methods.

**Figure 2:**
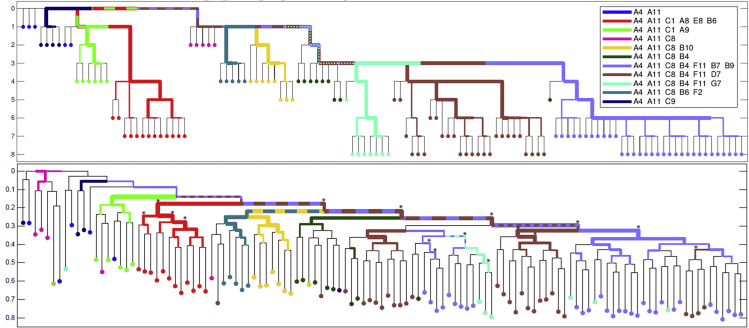
DU145 *ex vivo* tree comparison as reconstructed by the duplex MIPs pipeline (A) The *in vitro* reference tree, colors assigned by shared ancestors labeled by sampling wells prefix, y axis indicated sampling generations. (B) The *ex vivo* lineage tree as reconstructed by duplex MIPs data by using the FastTree2 algorithm and uniform transition probabilities. Hypergeometric tests are carried out for each internal branch to assess whether subtree leaves are enriched for a given cell population; the results are reflected in the highlighting and width of edges. Edges reproduced with significant support (TBE > 70%) in bootstrap analysis of the duplex MIPs data are annotated with asterisks. The y axis indicates relative depth when the tree is rooted at the median of the A4_A11 group, which is closest to the real root and therefore used for its approximation.

As first proof of concept for the duplex MIPs lineage system, we reproduced a known lineage history of SCs from a previously developed DU145 *in vitro* benchmark tree (Biezuner et al., 2016) (Figure 2, DU145 *in vitro* tree is discussed in STAR Methods). Briefly, an SC from the DU145 human male prostate cancer cell line has been clonally cultured; iteratively SCs were picked from the previous culture and clonally cultured to generate an *in vitro* tree covering nine generations, each cultured for 12 to 15 cell divisions. In each iteration, SCs were picked using CellCelector, annotated with their plate/well coordinates in the lineage tree, and whole-genome amplification (WGA) products were prepared. The same WGA products produced by Biezuner et al. (2016) were subjected to analysis using a duplex MIPs panel (OM6, panel details in Data S1, coverage and mutations in Data S2 and Figure S4), encompassing ∼12,000 STR targets. After the library's sequencing by Illumina NextSeq, the raw data were subjected to tailored analysis in our computational pipeline (see sections “integrated bioinformatics database management system” and “tree reconstruction parameters” in STAR Methods). Here and in all other experiments presented, the same tools and parameters were used: mapping a custom reference of STR variations by using bowtie2, genotyping stutter profiles by using R&B method (Raz et al., 2019), coarse assignment of allelic identities, and finally parsimony-based phylogenetic reconstruction by using FastTree2 (Price et al., 2010). The results demonstrate imperfect but accurate reconstruction of the expected reference topology. Most of the major reference splits in this validation experiment are significantly supported in bootstrap analysis (Figure 2, significance marked at transfer bootstrap expectation (TBE) >70% (Lemoine et al., 2018).

**Figure 3:**
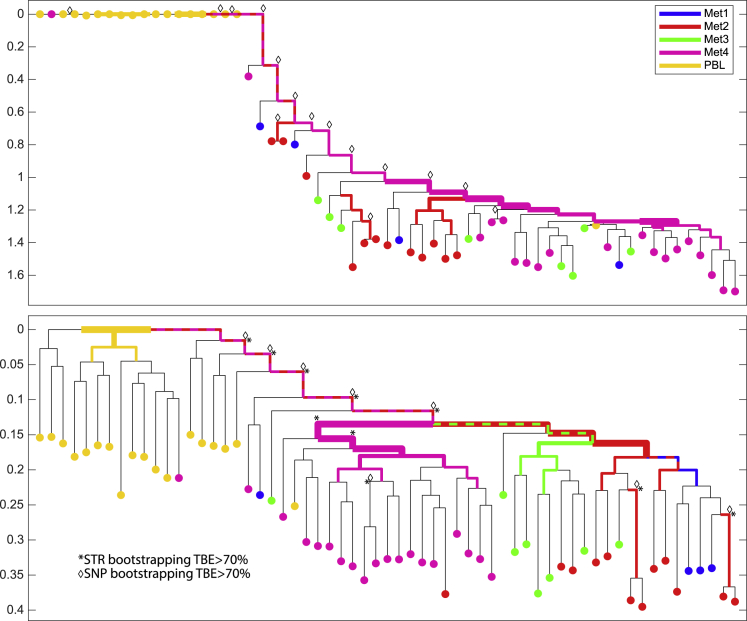
Cell lineage reconstruction of SCs from melanoma metastases and PBLs from the patient YUCLAT (A) SNV-based reconstruction dendrogram of YUCLAT, presenting significant separation (TBE > 70%, annotated by diamond symbols) of the expected separation of cancer cells from the PBLs. (B) STR-based reconstruction dendrogram of YUCLAT, presenting significant separation (TBE > 70%, annotated by asterisks) of the Met4 samples on top of the expected separation of cancer cells from the PBLs. In addition to the bootstrap analysis performed independently for the two trees, annotated by diamonds in (A) and asterisks in (B), when significant values were found, the SNV-based support is also annotated on top of the STR-based reconstruction in (B), emphasizing the refinement provided by STRs. Hypergeometric tests are carried out for each internal branch to assess whether its subtree's leaves are enriched for a given cell population; the results are reflected by the color and width of the edges. The y axis indicates relative depth when the tree is rooted at the median genotype of the PBL group, under the assumption that it would best approximate the true zygote root.

Consequently, in order to show the potential of duplex MIPs for SC lineage reconstruction of real human clinical cases, we opted to reconstruct the lineage of SCs sampled from a melanoma patient termed YUCLAT (Krauthammer et al., 2015). Five sample groups were obtained from the patient: normal peripheral blood lymphocytes (PBLs), skin metastases (Met 1, Met 2, and Met 3 recurring at the patient's right scapular area excised several months apart, and a more distant metastasis 4, from an axillary lymph node). Primary tumor samples were not available. The cells were processed by our duplex MIPs platform by using a panel that covers a similar set of ∼12,000 STR targets as earlier, with the addition of cancer-related SNV targets and patient-specific SNVs based on previous bulk exome sequencing data of the patient YUCLAT (Figure 3, panel OM6 andOM7 panel details in Data S1; coverage and mutations in Data S2 and Figure S4). The reconstructed STR lineage tree demonstrated an effective *in vivo* separation, validating the expected groups by near-perfect separation of the healthy samples (PBL) from the metastatic ones (Met 1–4). The same separation can also be validated utilizing known melanoma and patient-specific SNV mutations that are captured by the same MIP panel (Figure 3A). Significantly, the STRs derived information for those cells that goes further than the healthy/metastatic separation, refining the clusters and showing that Met4, sampled from an axillary lymph node, is clustered separately from the three other metastatic groups sampled from the patient's back (Figure 3B).

**Figure 4:**
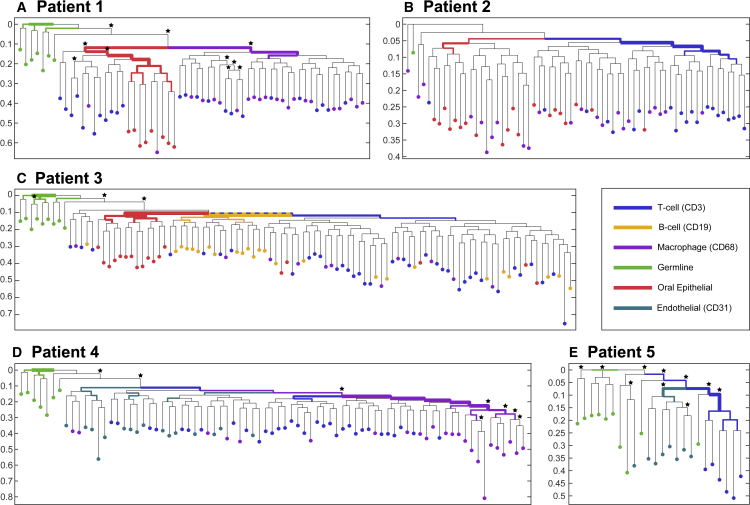
Cell lineage reconstructions of healthy cells Cell lineage reconstruction of healthy cell populations showing significant separation (TBE > 70%, annotated by asterisks) of OECs in patient 1 and of endothelial cells in patients 4 and 5 from the hematopoietic populations. This is expected by our current understanding of embryonic development, marking the separation of the embryonic germ layers. As this separation is not necessarily clonal (a SC gives rise to a germ layer), mixed clusters can also be observed. Hypergeometric tests are carried out for each internal branch to assess whether subtree leaves are enriched for a given cell population; the results are reflected by highlighting and width of the edges. The y axis indicates relative depth in all the patient trees and the trees are rooted at the median genotype of the germline group (bulk samples), under the assumption that it would best approximate the true zygote root.

Finally, although cancer cell lineages can be tackled by assessing various DNA sequence alterations, including SNVs, CNVs and STRs, due to genetic and genomic instability, healthy cells display normal mutation rates, rendering their lineage reconstruction significantly more challenging. In order to test if we can recover the lineage history of healthy cell populations and even trace embryonic cell lineage commitment, we subjected cells of hematopoietic (CD3+, CD19+, CD68+), endothelial (CD31+), and epithelial (oral epithelial cells [OECs]) origin (see “healthy human samples” in STAR Methods) to WGA and MIP analysis using the patient-generic duplex MIPs panel (OM6, panel details in Data S1, coverage and mutations in Data S2 and Figure S4). For WGA, we used the Ampli1-protocol (Klein et al., 1999, 2002), which was found superior for SC lineage tree reconstruction compared with other methods (Biezuner et al., 2017). Indeed, we demonstrate that this panel can identify mutations supporting the separation of embryonic germ layers in multiple patients (Figure 4); for example, significant “pure” clusters can be seen in patient 4 (macrophages) and in patient 5 (T cells), whereas significant mixed clustering can be seen in patient 1 (T cells and epithelial cells in the left cluster and T cells and macrophages on the right).

In complex organisms such as zebrafish, mouse, and human, coalescence of cell lineage trees is highly challenging. We have developed a scalable and cost-effective cell lineage discovery platform with low initial costs (comparison with other STR lineage platforms is discussed in STAR Methods) supported by a tailored bioinformatics analysis and database management system (integrated bioinformatics DBMS is discussed in STAR Methods). By applying this platform to various types of human cells, we demonstrate that tens of thousands of hyper-mutable STR targets in SC WGA DNA could be efficiently acquired, enabling lineage relations among these cells to be discovered.

After generating a proof of concept by using single cancer cells cultured over several generations, we refined an SNV-based cell lineage tree of a melanoma patient by applying our STR-based approach. In this experiment, the distant anatomical location of a lymph node metastasis was reflected by a distinct cluster in the tree structure obtained by STR but not by SNV analysis. Pushing the envelope further, we successfully applied the approach to single non-cancer cells of different types. In all five cases (Table S1), we obtained a non-random clustering of hematopoietic cells either versus oral epithelial or versus endothelial cells, which support early developmental separation of these populations. Ectoderm-derived OECs and mesoderm-derived cells separate at gastrulation, whereas mesoderm-derived endothelial cells and hematopoietic cells are derived from the hemangioblast during embryonic vasculogenesis (Risau and Flamme, 1995). Vascular regeneration, tissue maintenance, and cancer angiogenesis are currently thought to be driven by vascular endothelial stem cells from pre-existing organ vessels and not from bone marrow-derived endothelial precursor cells (Naito et al., 2016; Wakabayashi et al., 2018). In contrast, only in two patients (patients 1 and 4), we noted a separation of T cells (CD3) and macrophages (CD68). Given that CD68+ cells from patients 1 and 2 were isolated from peripheral blood and cells from patient 3 and 4 from lymph nodes, we could not establish a relation to the isolation source, i.e., between bone marrow-derived macrophages versus putative tissue resident macrophages (Ginhoux and Guilliams, 2016). We conclude that our current ability to separate cells of different lineages depends on the time point of separation from common ancestors, the number of cell divisions over a lifetime, and the mutation rate of each cell type. Somatic cells acquire mutations during each cell division over the course of an individual's life. Consequently, cells of a given type are likely to harbor multiple genetically distinct clones (groups of identical cells sharing common ancestry) (Risques and Kennedy, 2018). Of particular interest are mutations occurring early in embryogenesis or at early stages of tissue development/specification; these events are often present in a substantial proportion of daughter cells and thus have particular discriminatory characteristics. Clonal hematopoiesis leaves traces with the accumulation of somatic mutations in hematopoietic stem cells and their clonal expansion through the lifetime of patients (Acuna-Hidalgo et al., 2017; Busque et al., 2018; Jaiswal et al., 2014; Zink et al., 2017), and this might appear as conflicting annotation on the tree.

The duplex MIPs platform reduced the cost per cell as much as eight times compared with the AA pipeline (Table S2, incubation optimization). The duplex MIPs platform can scale up to 100,000 targets while keeping the capture cost almost the same. However, with AA pipeline for larger panels, the cost increases dramatically due to the cost of primer synthesis and limited multiplex capability, requiring extra AA chips (Figure S1I). The duplex MIPs pipeline introduces less *in vitro* STR stutter noise equivalent to 15–20 cycles of amplification (Figure S1F), and this in turn allows for confident genotyping with coverage as shallow as 5× compared with 30× for AA pipeline (Figure S1H). The genotypes from both pipelines correlate with 0.994 (Figure S1G). With 65% of the haplotype AA loci that are available in the duplex MIPs panel for DU145 *ex vivo* samples, similar reconstruction resolution was demonstrated by using the duplex MIPs system compared with AA. This asserts the reproducibility of STR-based lineage reconstruction and supports the relaxation of the strict male-patient X chromosome policy of the AA system without loss of accuracy. For the YUCLAT melanoma cancer patient (Figure 3), AA pipeline could only tell apart Met 4 from healthy PBLs; with the current platform, we showed a finer separation among the different metastasis groups on top of the separation from the PBL group.

Regarding the STR *in vivo* mutability, *in vitro* stability, and diversity in the human population, naturally occurring STR loci in the human genome vary in their mutability based on their repeat unit length, number of repeats, and GC content of the repeating unit. Although it is tempting to go after extremely mutable STRs such as poly G or CG repeats, we have previously shown those are inefficiently amplified *in vitro* (Raz et al., 2019). Furthermore, such loci are typically found in shorter lengths in the human genome (fewer repeats) and mutate often during *in vitro* amplification and even during the sequencing process itself.

While attempting to push the envelope ourselves, we found AC repeats to be a “sweet spot” in this *in vivo* mutability versus *in vitro* stability tradeoff with the current technologies and based most of this work on such loci. With further improvements mentioned in the discussion, mono-repeats could become favorable targets for cell lineage reconstruction.

Estimating on the basis of the cultured cell lineage tree (prostate cancer cell line DU145), the average mutation rate per generation was ∼10^−3^. This was determined for the loci on the haplotype X chromosome. Typically, we find STR-based phylogenetic decisions are supported by multiple independent mutating loci, limiting the effect of possible reversal mutations.

As such highly mutable targets, the mono- and di-repeat STRs make a noisy source of mutations. However, coalescing mutations from multiple independent loci strengthen their implied clustering claim. Such events can be systematically measured and spotlighted when bootstrap analysis consistently finds the same clusters in randomized reconstructions that leaves out about a third of the loci at every round.

An additional layer of noise is caused by insufficient heterogeneity in STR targets between its two alleles. We took two approaches: (1) choose STRs from X and Y chromosomes and male donors; (2) separate biallelic loci with more than three difference units of STRs. Although many loci show lesser heterogeneity, and assertions of maternal/paternal allelic identities cannot be confidently made, we found that such loci still contribute information to the phylogenetic reconstruction. One obvious artifact is longer edges between the leaves of the tree and their immediate ancestors, but we found both in simulations and with experimental data that the clustering accuracy is improved.

Although the sample throughput is not as high as droplet-based approaches like the mitochondria heteroplasmy-based lineage tracing system (Ludwig et al., 2019), our pipeline can be applied to both cells and nuclei that lack mitochondria. STR-based lineage holds the promise to reach single-cell-division resolution with further improvements. Evidently, technical improvements will also have an impact on faithful cell lineage reconstruction, including (1) reduction of the artificial noise introduced during WGA. This can be achieved by reaction volume reduction (Gole et al., 2013), by improvement of SC WGA increasing the uniformity of amplification across the genome (Chen et al., 2017), utilization of UMIs, or ideally the development of a direct MIP pipeline bypassing the WGA step. (2) Acquisition of higher-mutability STRs, such as mono-repeats, would improve performance in cases of fewer cell divisions and lower mutation rate. (3) Duplex MIPs targeting joint SNV-STRs regions within one amplicon can be designed to aid biallelic STRs genotyping. Improved haplotyping strategies can be implemented to harness this additional information (Gymrek et al., 2017). (4) Selective targeting of heterozygous STRs with significant difference in repeat numbers, filtering out close biallelic loci like (AC)X14, (AC)X15. To do this, a more accurate STRs annotated human reference genome is needed. (5) An improved loci selection during the panel planning might also allow higher success rates and uniformity, thus reducing the minimal sequencing coverage required per sample (sample sequencing coverage is discussed in STAR Methods). Uniformity can be also improved by optimization of the MIP protocol (e.g., by either empirical optimization of the capture reaction or optimized design of capture probes). Finally, cell states, SC spatial information, and phenotypes can be integrated into the cell lineage tree, which might be of significant interest for questions related to immune diseases, cancer, and tissue regeneration, among others (Ji et al., 2020). Thus, our platform will help study human development and cellular processes in both health and disease.

### Limitations of the study

Poor quality of whole-genome amplified genomic DNA will not work well in this protocol. The more uniform and representative the WGA, the better results this protocol can achieve.

## STAR★Method

### Key resources table

**Table:** 

REAGENT or RESOURCE	SOURCE	IDENTIFIER
**Chemicals, peptides, and recombinant proteins**		

Betaine solution	Sigma	Cat#5MB0306 1VL
Phusion High-Fidelity DNA Polymerase	NEB	Cat#NEB-M0530L
Ampligase 10X Reaction Buffer	EPICENTRE	Cat#A1905B
Ampligase DNA Ligase W/O Buffer	EPICENTRE	Cat#A3210K
Exonuclease I (E.coli)	NEB	Cat#M0293L
Exonuclease III (E.coli)	NEB	Cat#M0206L
RecJf	NEB	Cat#M0264L
Exonuclease T	NEB	Cat#M0265L
T7 Exonuclease	NEB	Cat#M0263L
Lambda Exonuclease	NEB	Cat#M0262L
NEBNext Ultra II Q5 MasterMix	NEB	Cat#M0544L
MinElute PCR Purification Kit	QIAGEN	Cat#28006
Qubit® dsDNA HS Assay Kit	ThermalFisher	Cat#Q32854
Agencourt Ampure XP Beads	BeckmanCoulter	Cat#A63881
2% Agarose, dye-free, BluePippin, 100 - 600,	Sage	Cat#BDF2010
TapeStation Screen Tap	Aglient	Cat#5067-5582
TapeStation Reagents	Aglient	Cat#5067-5583
MiSeq Reagent Kits v2	Illumina	Cat#MS-102-2002
MiSeq Reagent Nano Kit v2 (300-cycles)	Illumina	Cat#MS-103-1001
NextSeq 500/550 High Output Kit v2.5 (300 Cycles)	Illumina	Cat#20024908

**Deposited data**		

Sequencing data	ArrayExpress	.

**Experimental models: cell lines**		

DU145 cell line	ATCC	DU 145ATCC® HTB-81™

**Oligonucleotides**		

Oligopool	GeneScript	See supplemental informationfiles

**Software and algorithms**		

Clineage	github	https://github.com/shapirolab/clineage

**Other**		

selected STR loci	This Paper	OM6, OM7, OM9

### Resource availability

#### Lead contact

Further information and requests for resources and reagents should be directed to and will be fulfilled by the lead contact, Ehud Shapiro, ehud.shapiro@weizmann.ac.il.

#### Materials availability

This study did not generate new unique reagents.

#### Data and code availability

Sequencing data have been deposited with ArrayExpress access number E-MTAB-6411.

All the codes of cell lineage analysis tools mentioned in the text are freely available under the GNU General Public License v2.0 at https://github.com/shapirolab/clineage.

### Experimental model and subject details

#### DU145 *in vitro* samples

Single cells (hereby SCs) from DU145 human male prostate cancer cell line was separated via an automated cell picking device (CellCelector, ALS) and cultured to generate a nine-level *ex vivo* tree. SCs were seeded in separate microwells and underwent clonal expansion. Then, repeatedly, SCs were picked from the clonal microwells, seeded separately in new microwells, and expanded. Overall, SCs were sampled nine times, every 12-15 cell divisions. WGA DNA of these single cells was prepared using a modified RepliG Mini protocol as described previously (Biezuner et al., 2016).

#### WGA DNA of single cell from a melanoma patient

YUCLAT metastatic melanoma cells and Peripheral Blood Lymphocytes (PBL) were collected from a 64-yr-old male patient by the Specimen Resource Core of the Yale SPORE in Skin Cancer, with the participant's signed informed consent according to Health Insurance Portability and Accountability Act (HIPAA) regulations with a Human Investigative Committee protocol as described (Krauthammer et al., 2015). For Single cell DNA WGA preparation, the RepliG protocol was applied as described previously (Biezuner et al., 2016).

#### Healthy human samples

Cell of hematopoietic origin (T cells, B cells, macrophages) and endothelial cells were isolated from various fresh, non-fixed tissues (blood, lymph nodes) sent for analysis to the Institute of Experimental Medicine and Therapy Research, University of Regensburg. From some patients, buccal swabs and peripheral blood were also obtained. The study was approved by the ethics committees of the University Regensburg and Tübingen (07-079, 18-948-101, 535/2016B02). Written informed consent was obtained from all patients.

#### Preparation of adhesion slides for single cell isolation from human patient samples

##### Lymph nodes

Single cell suspension from lymph nodes were prepared as previously described (Ulmer et al., 2005, 2014). Briefly, lymphatic tissue was cut into 1-mm pieces with a sterile scalpel and disaggregated mechanically into a single-cell suspension using the Medimachine (DAKO) equipped with sterile cartridges holding rotating knives (Medicons, DAKO), washed with HBSS (Life Technologies) and centrifuged for 20 min at 1000 g and room temperature (RT) on a density gradient consisting of a 60% Percoll solution (Amersham). Mononuclear cells were isolated from the interphase, washed with PBS (300g, 10 min, RT), resuspended in PBS and counted. Subsequently, cell density was set to 10^6^ cell/ml in PBS and the resulting cell suspension was transferred onto adhesion slides (Thermo Fisher Scientific) at a density of 10^6^ cells/side (0,33 x 10^6^ cells/spot). After sedimentation of cells for 1 hour at RT, residual PBS was discarded and slides were air-dried overnight at RT and stored at -20°C.

##### Metastasis

The metastatic tissue was put in Basal medium consisting of DMEM/Ham’s F12 Medium with 10mM Hepes, 1% penicillin-streptomycin (all from Pan Biotech GmbH) and 1% BSA (Sigma Aldrich) and cut into smaller pieces. To obtain single cell suspension, tissue pieces were enzymatically digested with collagenase (Sigma Aldrich), hyaluronidase (Sigma Aldrich) and DNaseI (Roche) at a final concentration of 0.33 mg/ml, 100 μg/ml and 100 μg/ml, respectively, for 20-40 minutes at 37°C. Next, samples were further disaggregated by pipetting and transferred into a 50 ml tube. Following a washing step with PBS (300g, 10 min, RT), the cell pellet was resuspended in pre-warmed (37°C) Trypsin 0.05%/EDTA 0.2% in PBS (Pan Biotech) and treated for five minutes at RT. Digestion was stopped by adding 5 ml of Basal medium supplemented with 10% FCS (Pan Biotech). Next, cells were filtered using a 40μm cell strainer (Becton Dickinson GmbH) and washed with 14 ml PBS (300g, 10 min, RT). The cell pellet was re-suspended in PBS (10^6^ cells/ml) and the cell suspension was transferred onto adhesion slides at a density of 10^6^ cells/side (0,33 x 10^6^ cells/spot). Following sedimentation of cells on the slide (incubation 1 hour at RT), residual PBS was discarded, slides were air-dried overnight at RT and stored at -20°C.

##### Buccal swabs

Oral epithelial cells (OECs) were isolated by rubbing a sterile cotton swab on the patient’s buccal mucosa. The cotton swab was stirred in 1ml PBS and the obtained cell solution was transferred onto adhesion slides without prior counting. Following sedimentation of cells for 1 hour at RT, residual PBS was discarded and slides were air-dried overnight at RT and stored at -20°C.

##### Peripheral blood

Peripheral blood mononuclear cells (PBMCs) were washed with PBS (CellSave tubes) or Hanks solution (Biochrom AG, EDTA vacutainers) and centrifuged (200 g, 10 min, 4ºC). The cell pellet was diluted in Hanks solution and centrifuged (1000 g, 20 min, 4ºC) on a 60% Percoll (Amersham) density gradient. The interphase containing PBMCs were collected, washed in PBS (200 g, 10 min, 4ºC), resuspended and counted. PBMCs isolated from CellSave tubes were transferred onto adhesion slides at a density of 0.5x10^6^ cells/slide (0.17 x 10^6^ cells/spot). Following sedimentation of cells on the slide (incubation 1 hour at RT), residual PBS was discarded, slides were air-dried overnight at RT and stored at -20°C. PBMCs isolated from EDTA-vacutainers were cryo-conserved in 90% FCS and 10% DMSO (Applichem) in liquid nitrogen.

#### Isolation of single cells from adhesion slides

##### T cells, macrophages, endothelial cells

After thawing of adhesion slides (lymph nodes, PBMCs from Cellsave tubes, metastatic tissue) for 30 min at RT, unspecific binding was blocked using PBS/10% AB-serum (Bio-Rad) for 30 min at RT. To isolate CD3+ T cells, CD68+ macrophages and CD31+ endothelial cells, slides were stained with an anti-CD3 (cat. No. C7930, Sigma Aldrich), anti-CD68 (clone KP1, Agilent Dako) or anti-CD31 antibody (clone JC70A, Agilent Dako), respectively, for 60 minutes at RT. Rabbit IgG (cat. No. 0111-01, Southern Biotech) was used as isotype control for CD3 and CD31 immunostaining, IgG1k (clone MOPC 21, Sigma-Aldrich) for CD68 immunostaining. Secondary staining was performed for CD3 and CD31 with an AP-polymer anti-rabbit solution (ZUC031-006, Zytomed Systems) or for CD68 with an AP-polymer anti-mouse solution (ZUC077-100) for 30 minutes at RT. Slides were washed with PBS (3 times, 3 min) and developed using the BCIP-NBT detection system (Bio-Rad Laboratories) for 12 min followed by washing with PBS (3 times, 3 min). If slide availability was limited, slides were subjected to a CD3/CD68 double staining: following the incubation with primary antibodies (see above), cells were stained with goat anti-rabbit IgG-Alexa Fluor 555 (Thermo Scientific) and goat anti-mouse IgG-Alexa Fluor 488 (Invitrogen) for 30 minutes at RT. Positively stained cells were isolated from slides with a micromanipulator (PatchMan NP2, Eppendorf) and subjected to whole genome amplification (see below).

##### Oral mucosal epithelial cells

After thawing adhesion slides for 30 min at RT, OECs were identified based on morphologic criteria – diameter of 60-80μm with clearly smaller nucleus and an irregular shape- and isolated using a micromanipulator (PatchMan NP2, Eppendorf). Isolated cells were subjected to whole genome amplification (see below).

#### Isolation of single cells from frozen PBMCs

For isolation of single cells, cryo-conserved PBMCs were thawed in RPMI 1640 Medium (Pan Biotech) and 10% FCS, washed with PBS and counted. To identify single B and T cells, the single cell suspension was stained with anti-CD19 PE (clone HIB19, Biolegend) and anti-CD3 FITC-conjugated antibodies (clone HIT3a, Biolegend) for 15 min at 4°C, respectively. Unspecific antibody binding was blocked with PBS/10% AB-serum (Bio-Rad) for 5 min at 4°C prior to staining with CD19 or CD3. Cells were washed once with PBS/2% FCS/0.01% NaN3. Viability dye eFlour 780 (ebioscience) was used to enable discrimination of live and dead cells. CD19- and CD3-positive cells were isolated with a FACSAria cell sorter (BD Bioscience). Single cells were isolated from sorted populations using the micromanipulator (PatchMan NP2, Eppendorf) and subjected to whole genome amplification (see below).

##### Preparation of germline samples

Germline samples were prepared from bulk genomic DNA (gDNA) lymph node cells or PBMCs. gDNA was isolated using the DNeasy Blood & Tissue Kit (Qiagen, Hilden, Germany) following the manufacturer’s protocol. 200ng of unamplified bulk gDNA was used as template for library preparation with MIP-based probes and lineage analysis. In addition, 1ng and 10ng of genomic DNA were amplified (in three technical replicates for gDNA from the breast cancer patient and five technical replicates for gDNA from the melanoma patients) according to the whole genome amplification protocol (see below). The resulting samples were purified using the AMPure XP beads (bead to DNA ratio of 1.8X) following the manufacturer’s protocol.

### Method details

#### Whole genome amplification

Whole genome amplification (WGA) from single cells was performed as previously described (Klein et al., 1999, 2002). Following WGA, quality of the resulting products was controlled using a dedicated endpoint PCR assay (WGA-QC) (Polzer et al., 2014). Only DNA products with a genome integrity index (GII) of 3 and 4 were included in downstream analyses. As CD3+, CD19+, CD68+, CD31+ and OECs were isolated from tissues specimens of cancer patients, all cells were subjected to Ampli1TM LowPass Kit according to the manufacturer’s instructions (Menarini Silicon Biosystems). Only cells with a high quality balanced CNV-profile were considered for MIP-based analyses. Prior to MIP-based analyses, all WGA samples were re-amplified as described previously (Czyz et al., 2014). Every WGA product was re-amplified in two technical replicates. The temperature setting of the elongation steps included in re-amplification reaction was varied between the two technical replicates. One of the two re-amplification reaction was conducted with the temperature of the elongation step set to 65°C, while the other reaction was run with a corresponding setting set to 68°C. Once prepared both technical replicates were mixed in 1:1 ratio prior to further use. In addition, to eliminate residual oligonucleotides and reagents, pooled re-amplified samples were purified with AMPure XP beads with bead to DNA ratio of 1.8X (Beckman Coulter) following the protocol of the manufacturer.

#### Target specific duplex MIPs design and preparation

Hyper-mutable STRs were chosen based on the hg19 reference human genome annotation. The selection criteria were that the AC- and AG-type STRs should be longer than 10 repeats; the A- and G-type STRs should be longer than six repeats. SNVs targets were chosen from cancer related highly mutable regions or known cancer associated regions. Furthermore, additional criteria were applied during selection of the target regions: amplicons containing TTAA sequences were ruled out to comply with the design of the Ampli1 WGA kit and amplicons were selected to be ∼150bp in size.

Primer-3 based python script was used to design the targeting primers. Only top-scored primers were chosen as candidates for duplex MIPs precursor design. Additional filters were applied to the selected candidates: precursors harboring MlyI restriction site were ruled out; the total length of the precursors should not be longer than 150bp, which was the limit of the oligonucleotide synthesis provider.

Each duplex MIP comprises multiple functional elements (see below) ordered in the following sequence:

5’-[Mly1_F] + [FW_PRIMER_SEQ] + [UMI] + [Backbone] + [UMI] + [(RV_PRIMER_SEQ) in reverse-complement orientation] + [(Mly1_R) in reverse-complement orientation]-3`. The mentioned elements were designed as follows: Mly1_F:5’GTCTATGAGTGTGGAGTCGTTGC3`; Mly1_R:5’CTAGCTTCCTGATGAGTCCGATG3`; FW_PRIMER_SEQ, RV_PRIMER_SEQ: variable primers designed to chosen targets; UMI: 5’-NNN-3’ (N stands for randomized base over A, C, G, T); Backbone 5’-AGATCGGAAGAGCACACGTCTGAACTCTTTCCCTACACGACGCTCTTCCGATCT-3’

Ready-to-use duplex MIPS were generated from precursors by removing Mly1_F and Mly1_R with MlyI overnight digestion.

#### Duplex MIPs preparation

OM6 or OM7 pools (lists of the oligonucleotides comprised in each of the duplex MIP designs is listed in the Data S1 were synthesized by Custom Array (GenScript), Inc. The pools were diluted to a final concentration of 1ng/μl and used as templates. PCR primers (OM4_Mly_F:5`GTCTATGAGTGTGGAGTCGTTGC3`; OM4_Mly_R:5`CTAGCTTCCTGATGAGTCCGATG3`) were designed to fit the universal adaptors.

##### PreAmp PCR

Amplification of OM6 and OM7 precursor oligonucleotide pools was conducted in a 96 well plate by PCR reactions composed of the following reagents:

0.2ng/μl template, 0.3pmol/μl of each OM4_Mly_F and OM4_Mly_R primers, 1X KOD Polymerase MIX (Novagen, Toyobo) prepared according to manufacturers’ instructions, adding 0.5X SYBR green I (Lonza) . Final volume of each reaction was 45ul, typically 4-16 reactions were done per panel. The PCR was executed in the LightCycler 480 (Roche) using the following program: 95°C for 2 min (denaturation), 18 cycles of 95°C for 20 sec, 60°C for 10 sec, 70°C for 5 sec followed by elongation at 70°C for 5 min and cool down. Following the PCR, the reactions were pooled in sets of four and purified on one column each (MinElute PCR purification kit, Qiagen) according to the manufacturer's protocol, eluting in 45ul DDW. Concentration of the purified samples was measured using the Qubit dsDNA HS Assay Kit (Life Technologies). The purified PreAmp PCR product was diluted to 1ng/μl and served as a template for the next step (i.e. Production PCR).

##### Production PCR

Production PCR reactions (final volume 45ul) were done in a 96 well plate using the same recipe and the same program as in the PreAmp PCR, but running 12 cycles, typically 32-48 reactions were done per panel. The same purification procedure was executed: 4 reactions per purification column eluting in 45ul DDW. The purified products were merged and the concentration was measured using the NanoDrop spectrophotometer (Thermo Scientific). The pool of purified PCR products was diluted to ∼30ng/μl and subsequently processed in the next step.

##### MlyI digestion of the duplex MIP precursors

In order to cleave the adapter sequences and generate the active form of duplex MIPs, MlyI digestion was carried out by mixing 84ul of the diluted DNA (30ng/μl), 10μl of 10x NEB CutSmart Buffer (NEB) and 6U MlyI (final concentration of 0.6U/μl MlyI) in a 100ul reaction. Several reactions were performed, keeping 20μl of the undigested sample for quality control. The reactions were incubated in Biometra T3 thermal cycler (Biometra) at 37°C for 12 hours, inactivated at 80°C for 20 min and finally kept at 4°C. Subsequently, each reaction was purified using the MinElute PCR Purification Kit (Qiagen) and eluted in 25ul DDW.

##### Quality control of the duplex MIPs

All digested and purified products were merged and concentration measured using the Qubit dsDNA HS assay kit according to the manufacturer's protocol. The size of the digested product (∼105bp) and the undigested sample (∼150bp) was verified by running them on a TapeStation (Agilent) screentape (Figure S1A). If unwanted byproducts (represented by minor peaks in the TapeStation output plots) were detected in samples after digestion, BluePippin size selection method (Sage Science; 3% Gel Cassettes, 105bp tight mode) was used for further refinement of the MIPs with desired size (∼105bp).

##### Preparation of duplex MIPs working solution

Based on the average size of the duplex MIPs (105bp) and measured concentration, the purified digestion product was diluted to a final concentration of 80nM (80fmol/μl) (equivalent to 5.8ng/μl for OM6 and OM7 panels). This solution was used as a storage stock. The stock solution was further diluted 10 times to prepare a working solution (8nM) needed for the sequence capture experiments. Working solutions were stored at -20°C. The list of all major reagents mentioned above appears in Table S2.

#### Duplex MIPs-based targeted enrichment pipeline

##### Hybridization

Single cell WGA product concentration is generally 100-200ng/μl. To 2ul of single cell WGA DNA (200∼500ng) ,8ul of reaction mix was added with final concentration of 0.8fmol/μl duplex MIPs, 1X Ampligase Buffer and 0.9M betaine, in total volume of 10ul. The Hybridization was done using the following program:

100°C lid temperature, 98°C for 3 minutes, followed by a gradual decrease in temperature of 0.01°C per second to 56°C and incubated at 56°C for 17 hours.

Optionally, if the PCR machine cannot decrease the temperature as slowly as 0.01°C/second, an alternative strategy can be applied: 100°C lid temperature; denature at 98°C for 3 minutes; decreased by 0.1°C in a step-wise way, hold for 15 seconds for each step, until 56°C then incubated at 56°C for 17 hours.

##### Gap filling

The gap filling mix was prepared before the end of the hybridization, with final concentration of: 0.3mM each dNTP, 2mM NAD (freshly thawed from -80°C), 1.1M betaine, 1X Ampligase buffer, 0.5U/μl Ampligase and 0.8U/μl Phusion® High-Fidelity DNA Polymerase, 10ul/reaction and kept at 56°C in a heat block.

At the end of the hybridization, the plate was removed from the PCR machine to a 56°C heat block. 10μl of the gap filling mix were added to each well, carefully mixed by pipette, sealed tightly and quickly returned to the PCR machine at 56°C for 4 hours, followed by 68°C for 20 minutes and 4°C until next step.

Optionally, after the gap filling, the reaction plate can be stored at 4°C for up to two days.

##### Digestion of linear DNA

Digestion mix was prepared and 2ul/reaction were added with final concentration of: 3.5U/μl Exonuclease I, 18U/μl Exonuclease III, 4U/μl T7 Exonuclease, 0.4U/μl Exonuclease T, 3U/μl RecJ_f_, and 0.2U/μl Lambda Exonuclease. Mix well. The reaction plate was sealed, spun down and incubated at 37°C for 60 minutes, 80°C for 10 minutes and 95°C for 5 minutes. The reactions can be stored at -20°C for months after the digestion step.

#### Optimization of the duplex MIP target enrichment workflow

##### Selection of optimal ratio of template DNA and duplex MIPs in the target enrichment step:

Whole Genome Amplification (WGA) is a required step for most single cell genomics studies. However, the stochastic nature of WGA protocols can pose challenges for downstream analyses (Fu et al., 2015). Assuming that a limiting concentration of the probe could make up for underlying WGA biases, we sought to calibrate the ratio between duplex MIPs concentration and template DNA amount. For this purpose, variable amounts of template Hela DNA (range 0.01-2000ng) were hybridized with various concentrations of MIPs (ranging from 0.08 to 80nM). Performance of sequence capture was evaluated based on the outcome of the subsequent sequencing analysis. Samples with less than 8000 reads in total or under 4000 detected unique loci were regarded as outliers due to low coverage and were excluded from further evaluation. Conditions with 8-80nM of duplex MIPs probes and 250-500ng input DNA produced the most robust target capture efficiency (Figure S1B). Considering the yield of single-cell WGA reaction, we decided to use 8nM MIPs and 250-500ng single cell WGA DNA as the template DNA for our standard protocol.

##### Optimization of reaction times of the critical steps of the duplex MIP-based target enrichment protocol

Next, we tested the impact of duration of hybridization, gap filling and nuclease digestion (the three key steps of the duplex MIP-based workflow) on the performance of the target capture. To this end, hybridization was run for 2, 4 and 18 hours, gap filling was conducted for 1, 2 and 4 hours and nuclease digestion was executed for 1 and 2 hours. Variations of the protocol were compared in all by all combinations, amounting to 18 combinations, using HeLa genomic DNA (NEB) at a concentration of 200ng as template and 80nM duplex MIPs (OM6, Data S1). The best performing protocol was selected following sequencing and assessment of target capture efficiency. Among all tested conditions, the protocol with hybridization for 18 hours, gap filling for 4 hours and nuclease digestion for 1 hour proved to be the best and was used in all succeeding experiments. Following the sequencing of the enriched libraries at a sequencing depth of 10∼15 X, we found that under the optimized conditions ∼83% of the resulting reads successfully mapped to the target region and a similar percentage of targets represented in the panel could be successfully detected (Table S2, an average of 76% of the loci at an average coverage of 226K reads/sample).

##### Library size selection

In order to choose the proper library size, several ranges were chosen for comparison in the BluePippin (Sage Science) size selection step: broad range 240-340bp, narrow range 270-310bp and tight range 300bp, based on the designed amplicon size distribution. The sequencing results of 7 SCs WGA samples and one DNA bulk sample, revealed that both, the 300 and 270-310bp selection ranges had slightly better success rate (2∼3%) compared to the 240-340bp range; however, the 240-340bp size selection range stood out with more loci captured (8∼15%). Therefore, 240-340bp size selection range was chosen for future experiments (Table S2 size selection).

#### The combination of two independent panel OM6 and OM8

To test the feasibility of combine two independent panel of duplex MIPs, an independent panel of duplex MIPs OM8 (Data S1 and Figures S1C and S1D), no shared targets between OM8 and OM6, was prepared and tested with OM6. 8nM OM6 and 8nM OM8 were pooled by 1:1 and used to capture in a single reaction with a modified protocol where 59.7°C was used as Hyb and Gap steps instead of 56°C.

#### Sequencing library preparation

##### Sample specific barcoding PCR

Sample specific barcoding PCR was performed using dual-index barcoding primers and NEBNext Ultra II Q5 DNA Polymerase. The structure of the dual-index Illumina barcoding primers was previously reported Biezuner et al.(Biezuner et al., 2016).

I5-index-primer: AATGATACGGCGACCACCGAGATCTACAC [i5-8bp- index] ACACTCTTTCCCTACACGACGCTCTTCCG;

I7-index-primer: CAAGCAGAAGACGGCATACGAGAT[i7-8bp-index] GTGACTGGAGTTCAGACGTGTGCTCTTCCG; 2μl of the target enriched product was used as template, 0.5pmol/μl (final concentration) of each unique barcoding pair of dual-index Illumina primers for each sample, 1X NEBNext Ultra II Q5 Master Mix; 0.5X SYBER Green in a 20μl PCR reaction. The PCR was performed in a Roche 480 Light Cycler using the following program: 98ºC, 30 sec (denaturation), 5 cycles of 98°C for 10 sec, 56°C for 30 sec and 65°C for 45 sec; 17 cycles of 98°C for 10 sec, 65°C for 75 sec; followed by 65°C for 5 min (elongation); cool down.

##### Purification and pooling of barcoding PCR samples

The barcoding PCR product was cleaned by adding 0.8X (by volume) AMPure XP SPRI magnetic beads (Beckman Coulter) according to manufacturer’s manual and eluted in 38μl DDW. The cleaned PCR products were transferred by robot (Bravo, Agilent) to a 384 well plate and an equal volume of each of them pooled by Echo (Echo550, Labcyte). The pool was concentrated by MinElute column (Quiagen) to 35ul.

##### Size selection for diagnostic sequencing

The concentrated pool (30μl) was loaded on a 2% V1 cassette for BluePippin (Sage Science) size selection with setting range 240-340bp according to manufacturer’s protocol while 3μl of the concentrated pool was kept for later quality control. The size-selected elution was collected, cleaned by MinElute and eluted with 15μl DDW. The concentration was measured by Qubit dsDNA HS assay kit. The size distribution of the concentrated pool before and after BluePippin was checked by Tape Station dsDNA screen tape. The size-selected pool with a single peak around 300bp was used to prepare 12μl of 4nM (4fmol/μl) ready-to-run library for Illumina NGS.

##### Diagnostic sequencing

The ready-to-run library was sequenced on MiSeq Nano (MS-103-1001, Illumina) or Micro flow cell (MS-103-1002, Illumina) with 151x2 paired end run parameters according to manufacturer’s manual, the default sequencing primers were used.

##### Production sequencing

Based on the diagnostic sequencing results, a new pool was produced by Echo550 with a volume adjustment for each sample in order to equalize the reads. The pool was concentrated by MiniElute to 35ul and used to prepare a sequencing library as in step “size selection for diagnostic sequencing.”

The normalized library was sequenced on NextSeq 500) FC--404-2004Illumina) flow cell with 151x2 paired end, run parameters according to the manufacturer’s manual, the default sequencing primers were used.

#### Sequencing coverage

For the healthy human samples of Figure 4, a target coverage of 5M reads per cell was set in order to saturate coverage question and allow for accurate estimation of the required coverage. Considering the three main patients that had a substantial number of successful samples, we repeated the bootstrapping experiment with a range of computationally subsampled coverage values. The cases are compared against the tree reconstructed with full data, considering normalized triples distance as tree distance (TreeCmp(Bogdanowicz et al., 2012), Figure S3).

#### Duplex MIPs workflow timeline

The whole workflow of duplex MIPs pipeline took 5days from hybridization to data analysis, with roughly 3-hour hands on time (Figure S1E).

### Quantification and statistical analysis

#### Integrated bioinformatics Database Management System (DBMS)

We designed and implemented a scalable architecture of Cell Lineage Discovery Workflow DBMS for collaborative cell lineage studies. The system supports (i) Data storage and labeling that allows access to workflow data, including each donor’s data (anonymously) as well as samples’ details like origin, cell type, physical location, downstream handling, sequencing results and analysis steps; (ii) Jupyter integration for customized data mining and analysis; (iii) Tracking of workflow protocols, algorithms, sequencing and data analysis steps.

The design dissects both the biological and the computational workflow into atomic objects that are documented, referenced and stored for every experiment. Samples are documented using the web based graphical user interface, from individual to SC DNA resolution (Figure S2A). Processes such as targeted enrichment protocols, library preparation (Figure S2B) and NGS outputs are documented for all the cells. The NGS raw data (BCL files) is uploaded to the system and demultiplexed according to the documented sample information. After merging paired-end reads, each sample can be processed by either of the two STR-aware alignment pipelines developed in-house. The alignment is implemented for parallel execution on a computing cluster using the Dask distributed package. This results in the creation of millions of histograms depicting the STR length distribution for each cell-locus combination. The analysis is designed with fail-safe points at every step so that any underlying malfunction can be recovered, if needed, from prior computational steps. Those histograms are then processed by the genotyping module employing our STRs amplification stutter model (Raz et al., 2019). Individual genotyping results are aggregated for haplotyping, harnessing a population wide perspective to discern the two underlying alleles for each locus (Figure S2C).

Following haplotyping, bi-allelic cases are treated as two distinct mono-allelic loci, allowing for integration with unaware phylogenetic reconstruction tools. The system accommodates multiple distance based phylogenetic reconstruction algorithms (Price et al., 2010; Saitou and Nei, 1987) as well as other hierarchical clustering approaches. Reconstructed phylogenetic trees can be further processed by in-house adaptations of tree plotting tools as well as multiple methods for clustering assessment.

#### Tree reconstruction parameters

The same computational pipeline and set of parameters was used across all the experiments mentioned in the manuscript. A new mapping strategy was used for duplex MIPs sequencing data that improved computational efficiency. Reads were aligned against a custom reference genome of all possible STRs variations in the panel. Sequenced data with a minimal coverage of 10X reads was genotyped and a confidence threshold of 0.05 (correlation above 0.95) between the measured histogram and the reported model was set. The reconstruction based on these resulting genotypes was performed using the FastTree2(Price et al., 2010) algorithm with the mutation count distance matrix.

### Additional resources

Table S2 duplex MIPs pipeline optimization; related to Figure 1 and STAR methods. Data S1 panel details; related to Figures 2, 3, and 4. Data S2 mutation details; related to Figures 2, 3, and 4.

## Supplemental information

Document S1. Figures S1–S4 and Table S1

Table S2. Duplex MIPs pipeline optimization related to Figure 1 and STAR methods

Data S1. Panel details related to Figures 2, 3, and 4

Data S2. Mutation details related to Figures 2, 3, and 4

Document S2. Article plus supplemental information

## Footnotes

Supplemental information can be found online at https://doi.org/10.1016/j.crmeth.2021.100054.
