# Supplementary material for: Retrospective cell lineage reconstruction in humans by using short tandem repeats
Source: Cell Rep Methods. 2021 Jul 26;1(3):100054. doi: 10.1016/j.crmeth.2021.100054 (PMC8313865; doi:10.1016/j.crmeth.2021.100054)
Supplement: Document S1. Figures S1–S4 and Table S1 [file mmc1.pdf]

Cell Reports Methods, Volume *1*

Supplemental information

Retrospective cell lineage reconstruction in humans by using short tandem repeats

Liming Tao, Ofir Raz, Zipora Marx, Manjusha S. Ghosh, Sandra Huber, Julia Greindl-Junghans, Tamir Biezuner, Shiran Amir, Lilach Milo, Rivka Adar, Ron Levy, Amos Onn, Noa Chapal-Ilani, Veronika Berman, Asaf Ben Arie, Guy Rom, Barak Oron, Ruth Halaban, Zbigniew T. Czyz, Melanie Werner-Klein, Christoph A. Klein, and Ehud Shapiro

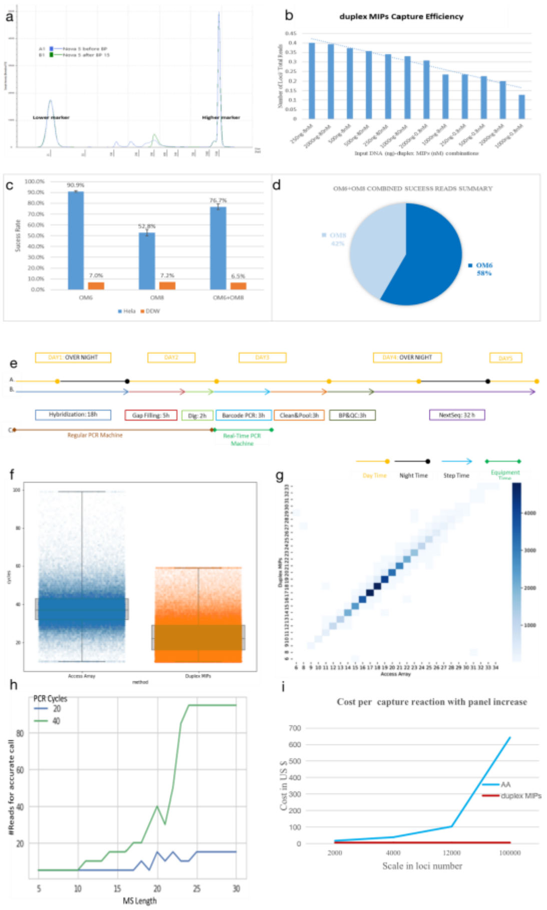

Figure S1: Duplex MIPs pipeline optimization and comparison with Access Array pipeline (AA pipeline) ; Related to Figure1 and Star Methods. a. Library Quality Control: Tape Station Electropherogram before and after Blue Pippin size selection run b. Comparison of target capture efficiency between different experimental conditions utilizing various MIP to template ratio. Efficiency of capture was evaluated based on the ratio of the number of loci detected to the number of total reads. Average value of two replicates for each test was used in the figure. c,d. The combination of two independent panel OM6 and OM8 | Hela bar (blue) for OM6 is average value of two replicates. Hela bar for OM8 is average value of three replicates. OM6+OM8 bar is average value of two replicates. DDW (orange) is negative control, no replicates. Pie chart is average value of two OM6+OM8 replicates value. *e.* Duplex MIPs workflow timeline (A). Day counts (B). Reaction step time count (C). Machine time count. f, Amplification cycles comparison as measured by fitted STR stutter noise model; g, Genotyping comparison of identical samples/loci, AA vs Duplex MIPs, correlation=0.994; h, Simulation analysis for the minimal number of reads required for accurate (less than one mistake in 1000 attempts) genotyping of AC microsatellite using AA protocol (estimated 40 amplification cycles) and using duplex MIPs protocol (estimated 20 amplification cycles); i, cost/scale projections of AA and duplex MIPs pipelines.

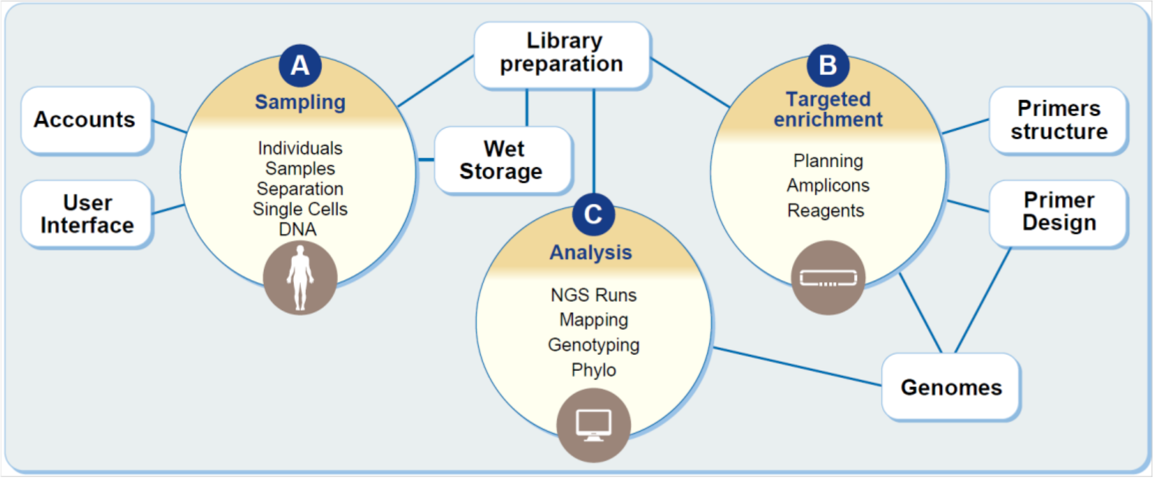

Figure S2 Outline of the integrated bioinformatics Database Management System; Related to Figure 1 and STAR methods. (a) Sampling: sampling documentation from patient to DNA, paired with User Interface for viewing, searching and documenting sampling components; (b) Targeted Enrichment: documenting target selection and probe design; (c) Analysis: steps from NGS raw data to Tree across multiple tools, versions and parameters. Paired with the Dask Distributed package for computing clusters.

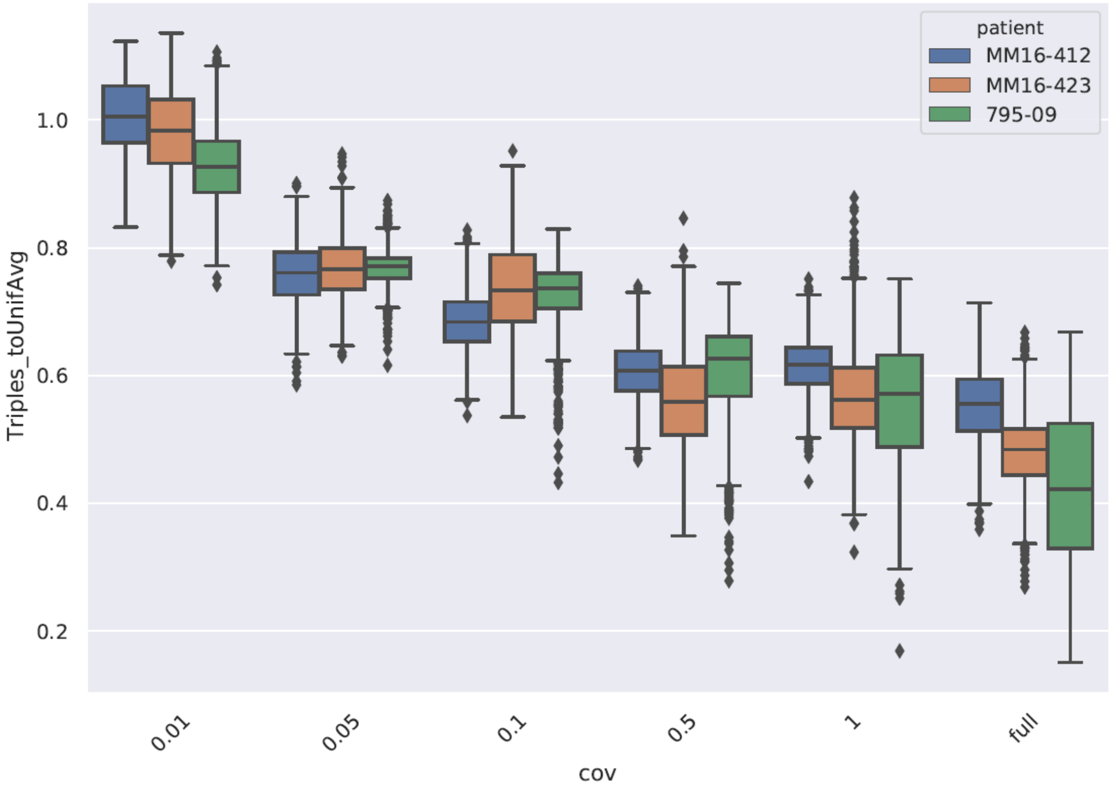

Figure S3: Repeated bootstrapping experiment with a range of computationally sub-sampled coverage values (x-axis, in million reads); Related to Figure 4 and STAR methods. The cases were compared against the tree reconstructed with full data, measuring normalized triples distance (y-axis).

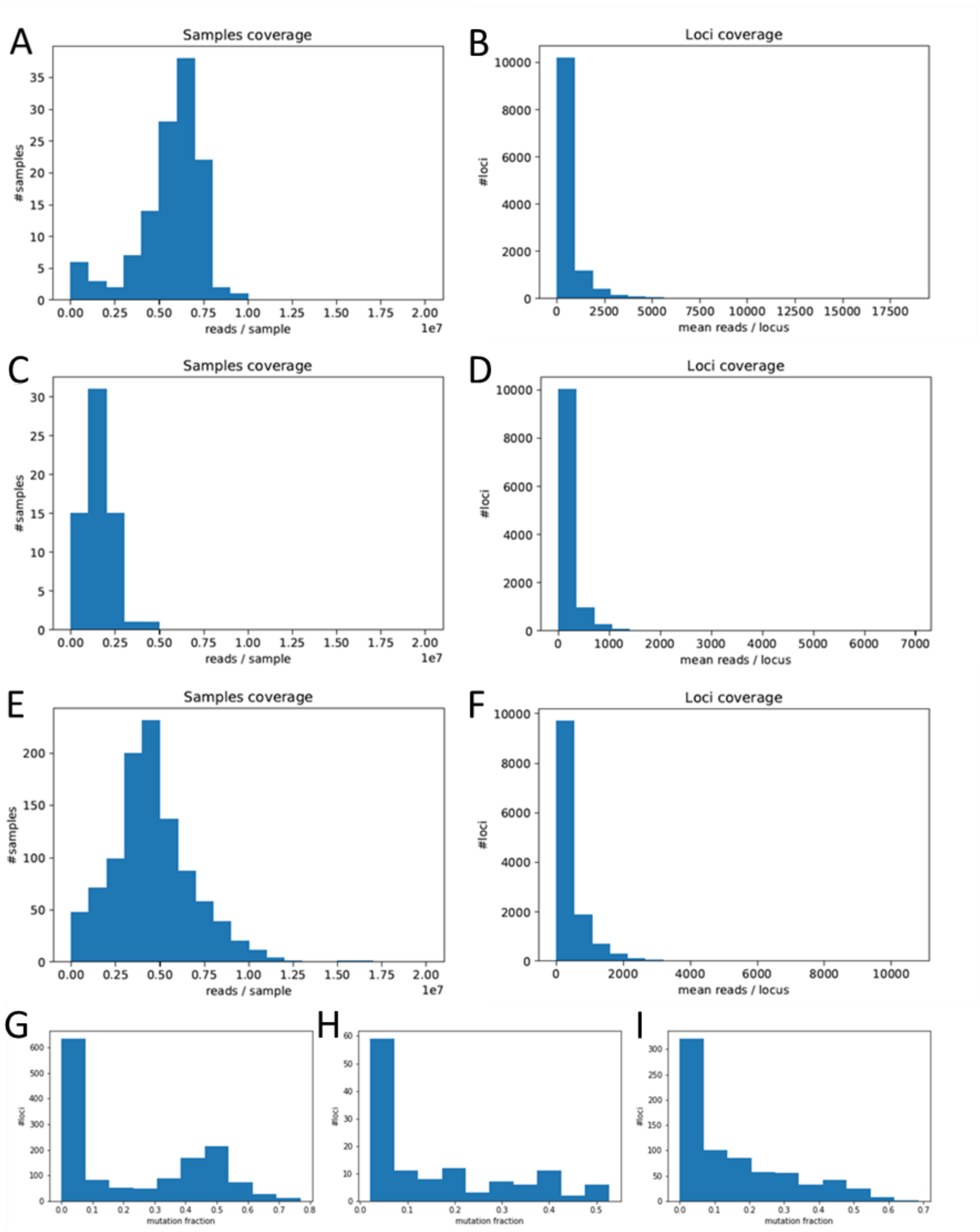

Figure S4: Reads coverage distribution and Mutated loci distributions of haplotype STRs; Related to Figure 2,3, 4; Reads coverage (by sample, by loci): (A, B), for Figure 2; (C, D), Figure 3; and (E, F) for healthy samples MM16-423's of Figure 4; Mutated loci distributions of haplotype STRs for Figures 2 (G), 3 (H) and 4 (I). Here only loci that were successfully genotyped in at least 30% of the samples are considered. If we consider for example loci that present a mutation in over 0.3 of the cells we consider 42% of the loci of (G), 23% of (H) and 20% of (I).

	Patient 1	Patient 2	Patient 3	Patient 4	Patient 5
**Gender**	Female	Female	Male	Male	Female
**Age at primary sampling**	62	46	59	64	79
**Germline (bulk)**	PB	PB	PB	PB	LN
**CD68+ Macrophages**	PB	PB	LN<br>Met	LN	LN
**CD3+ T cells**	PB	PB	LN<br>Met<br>PB	LN	LN
**CD19 + B cells**	--	--	PB	--	--
**CD31+ endothelial cells**	--	--	--	LN	LN
**Oral epithelial cells**	OM	OM	OM	--	--

LN: lymph node; PB: peripheral blood, Met: metastases; bulk: bulk genomic DNA**;** OM**:** oral mucosa

Table S1. Donor information related to Figure4.
